# An Improved U-Net Infrared Small Target Detection Algorithm Based on Multi-Scale Feature Decomposition and Fusion and Attention Mechanism

**DOI:** 10.3390/s24134227

**Published:** 2024-06-29

**Authors:** Xiangsuo Fan, Wentao Ding, Xuyang Li, Tingting Li, Bo Hu, Yuqiu Shi

**Affiliations:** 1 School of Automation, Guangxi University of Science and Technology, Liuzhou 545006, China; 100002085@gxust.edu.cn (X.F.); 221068348@stdmail.gxust.edu.cn (W.D.); 221077062@stdmail.gxust.edu.cn (X.L.); 221076985@stdmail.gxust.edu.cn (T.L.); 100000316@gxust.edu.cn (Y.S.); 2 Guangxi Collaborative Innovation Centre for Earthmoving Machinery, Guangxi University of Science and Technology, Liuzhou 545006, China

**Keywords:** U-Net, multi-scale feature fusion, haar wavelet transform, attention mechanism, infrared small targets

## Abstract

Infrared small target detection technology plays a crucial role in various fields such as military reconnaissance, power patrol, medical diagnosis, and security. The advancement of deep learning has led to the success of convolutional neural networks in target segmentation. However, due to challenges like small target scales, weak signals, and strong background interference in infrared images, convolutional neural networks often face issues like leakage and misdetection in small target segmentation tasks. To address this, an enhanced U-Net method called MST-UNet is proposed, the method combines multi-scale feature decomposition and fusion and attention mechanisms. The method involves using Haar wavelet transform instead of maximum pooling for downsampling in the encoder to minimize feature loss and enhance feature utilization. Additionally, a multi-scale residual unit is introduced to extract contextual information at different scales, improving sensory field and feature expression. The inclusion of a triple attention mechanism in the encoder structure further enhances multidimensional information utilization and feature recovery by the decoder. Experimental analysis on the NUDT-SIRST dataset demonstrates that the proposed method significantly improves target contour accuracy and segmentation precision, achieving IoU and nIoU values of 80.09% and 80.19%, respectively.

## 1. Introduction

Infrared target detection technology relies on distinguishing between the thermal radiation of the target and the background under an infrared imaging system to identify the target accurately. This is a crucial area of research in target detection [1]. In recent times, infrared detection systems have found widespread applications in various fields such as aerospace, precision guidance, early warning, public security, space debris detection, and military operations due to their advantages like effective concealment [2], all-weather operation, clear imaging, resistance to electromagnetic interference, and simple design [3]. Achieving high detection rates and real-time performance is essential for detecting small targets in practical scenarios. However, detecting small targets using IR detectors poses challenges such as small target size, weak signal in the IR image, poor image quality, and low contrast between the target and the background, making target detection more difficult [4]. Therefore, it is crucial to develop infrared small target detection algorithms that can meet practical requirements to further enhance the application of infrared technology in military and civilian sectors.

Traditional methods for detecting small targets in infrared imagery can be categorized as either Detect Before Track (DBT) or Track Before Detect (TBD). DBT involves first detecting the target in a single frame and then using target frame correlation for trajectory correlation to achieve detection. Common DBT algorithms include background prediction [5], mathematical morphology [6], transform domain filtering [7], and visual saliency-based methods [8]. While DBT is relatively straightforward, it struggles to provide accurate detection in low signal-to-noise ratio environments. On the other hand, TBD involves tracking trajectories in consecutive frames to identify potential target trajectories before confirming the target in a single frame for detection. Representative TBD algorithms include 3D matched filtering [9], projection transformation [10], dynamic programming [11], and particle filtering algorithms [12]. TBD methods are more complex and have limited real-time performance during detection. These traditional detection methods rely on prior knowledge of target characteristics to create operator models for detection, making them model-driven approaches.

In the past few years, advancements in computer hardware and artificial intelligence technology have led to significant advancements in deep learning for computer vision. Specifically, the use of convolutional neural networks has shown promise in detecting infrared small targets due to their strong feature extraction and model fitting capabilities. Unlike traditional algorithms for infrared small target detection, convolutional neural networks require a large amount of data to effectively learn the features of small targets, resulting in improved performance.

Target detection methods are usually categorized into one-stage [13] and two-stage detection methods [14]. The one-stage inspection method represented by the YOLO series [15] has excellent inspection speed and inspection accuracy, Suitable for infrared small target detection tasks. Wang et al. [16] combined DenseNet with the YOLO algorithm for infrared small target detection and utilized dense connectivity to fuse different levels of feature maps in the feature extraction process to better utilize shallow target information and reduce information loss. Cai et al. [17] on redesigning the backbone network of YOLO and reducing the number of downsamples, reducing the loss of target information, while using multiple paths to achieve feature fusion and improve the efficiency of feature extraction. An algorithm combined with deep learning was proposed by Huang et al. [18] Firstly, the spatio-temporal feature extraction network of YOLOv3 is utilized to detect the moving target, and then the traditional method of local contrast is utilized to detect the target quickly, which makes the algorithm achieve a good balance between accuracy and real-time performance. Dai et al. [19] combined the attention mechanism in YOLOv5 to enhance the feature extraction capability, while improving the loss function to optimize the training and help the model to converge, and then used a new prediction frame screening method to effectively improve the detection of small targets in infrared. Liu et al. [20] combined the optical flow algorithm with the YOLOv5, and the correlation information between frames is effectively utilized to overcome the problem of inaccurate extraction of motion trajectories of small targets. The YOLO algorithm is detected as an external rectangle, and many scholars use the data itself as a target with a high signal-to-noise ratio and a clear contour, which leads to a better target detection effect.

In addition to the detection of external rectangles, the pixel-level target description can better reflect the accurate target information, so many scholars use semantic segmentation to realize small target detection. Wang et al. [21] proposed a small target segmentation network with a two-branch structure, which uses two network structures to train the network separately and then combines the information from each structure, and this method can significantly reduce the leakage of small targets. Dai et al. [22] proposed the Asymmetric Context Modulation (ACM) algorithm, which reconsiders the feature fusion approach with the attention mechanism, supplementing the bottom-up modulation path to enable the network to embed finely-detailed low-level context into high-level features, and then employing a point-by-point channel attention module to highlight the features of a small infrared target. Li et al. [23] proposed the Dense Nested Attention Network (DNA-Net), which enables repetitive interactive fusion of feature information through densely nested interaction modules to retain small target information in the deep network while using channel and spatial attention mechanisms to further enhance target features. Wu et al. [24] nested a small U-Net into a larger U-Net to achieve multi-level and multi-scale representation learning of goals. To solve the problem of small target features being weakened in the deep layer of CNN, Li et al. [25] proposed an infrared low-level network (ILNet), which pays more attention to the underlying information, utilizes a feature fusion module to fuse the more important underlying information into the deep layer, and adds a dynamic one-dimensional aggregation layer to dynamically adjust the aggregation of the low-dimensional information according to the number of input channels. Balancing leakage detection and false alarms is an important issue in infrared small target segmentation, Wang et al. [26] proposed a conditional generative adversarial network (MDvsFA-cGAN) model consisting of two generators and one discriminator, which divides the reduction of leakage detection and false alarms into two sub-tasks to be given to two adversarial-trained models to deal with, which can be optimally balanced after adversarial learning and significantly improve the detection performance.

Infrared images often present challenges for detecting small targets due to their lack of texture features, weak signals, and susceptibility to noise. To address these issues, the target detection network must not only preserve target detail information but also extract multi-scale and multi-dimensional semantic information effectively. This enables the model to better understand both the target and its surrounding environment, leading to more efficient feature utilization. In this research, a new approach called MST-UNet is introduced, which enhances the U-Net model by incorporating multi-scale feature decomposition and fusion and an attention mechanism. The Haar wavelet transform is used to downsample feature maps and reduce information loss, while a multiscale feature fusion module is designed to capture contextual information at different scales. This enhancement improves model performance without increasing parameters or computational complexity. Additionally, a triple attention mechanism is employed to emphasize important information across various dimensions and further refine the feature representation.

The main contributions of this paper are as follows:

1. Instead of maximum pooling, downsampling based on haar wavelet transform is used to reduce the information loss caused by pooling computation and retain more detailed information.
2. A multi-scale feature fusion module is developed as the fundamental building block of the network to capture multi-scale contextual information through cavity convolution with varying cavity rates, enhancing the sensory field and boosting feature expression.
3. Following multilevel feature fusion within the decoder architecture, a triple attention mechanism is employed to enhance the utilization of multidimensional information, enabling the decoder to recover more feature details and enhance target segmentation accuracy.
4. Results from experiments conducted on the NUDT-SIRST dataset demonstrate that the proposed method in this study achieves superior segmentation outcomes, surpassing other algorithms in terms of Intersection-over-Union (IoU), Normalized Intersection over Union (nIoU), Probability of detection (Pd), and False-alarm Rate (Fa), providing a more accurate description of the target contour.

## 2. Data and Methodology

### 2.1. Experimental Data

**Figure 1:**
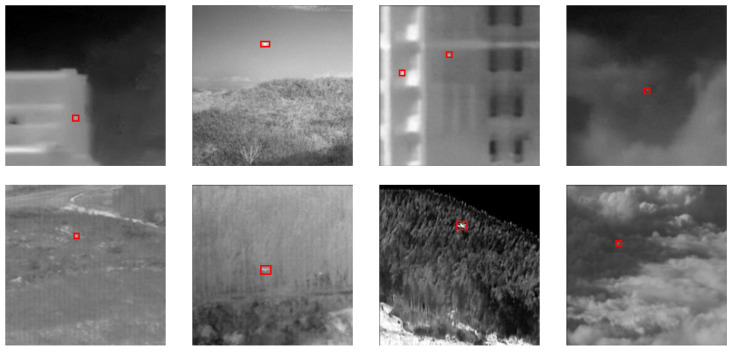
NUDT-SIRST datasets.

The NUDT-SIRST dataset was developed by Li et al. in 2022 [23]. It is capable of evaluating the performance of CNN-based research methods under multiple target types, target sizes, and different clutter backgrounds, as shown in Figure 1. which contains five scenarios including city, field, highlights, ocean, and clouds, with a total of 1327 images, The training and test sets were divided according to the ratio of 6:4, with 796 pictures in the training set and 531 pictures in the test set, the pictures were uniformly resized to 256 × 256 size for training and testing.

### 2.2. MST-UNet Overall Structure

**Figure 2:**
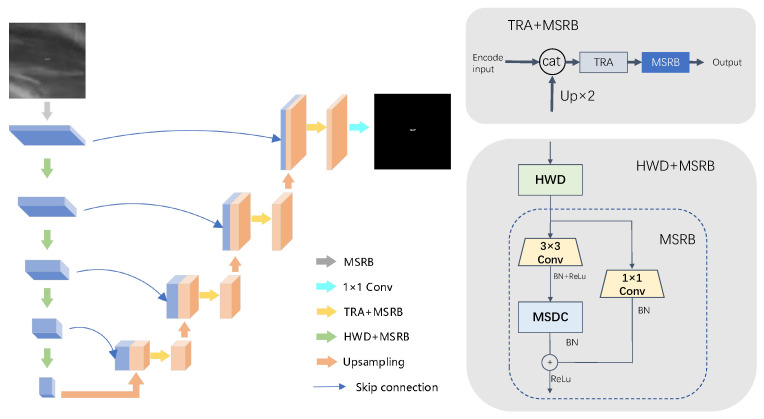
MST-UNet overall structure diagram, which utilizes a symmetrical encoder-decoder structure, the encoder structure contains four Haar Wavelet Transform downsampling structures (HWD) and five multi-scale residual modules (MSRB) for feature extraction. The decoder structure consists of four upsampling, triple attention mechanism (TRA), and multi-scale residual module for feature recovery. Fusion of feature maps at the same level of encoder and decoder using jump connections. The final prediction is made by a 1 × 1 convolution.

In this paper, based on the U-Net structure, we propose an MST-UNet network architecture based on multi-scale feature fusion and attention mechanism, its overall structure is shown in Figure 2. The overall architecture of MST-UNet uses a symmetric structure based on encoders and decoders, and utilizes hopping connections to fuse different levels of features in the encoders and decoders. Its basic constituent structures include Multiscale residual block (MSRB), Haar wavelet transform downsampling (HWD), triple attention mechanism, and transposed convolution. The input image size for this network structure is 256 × 256 × 3, and a total of five multiscale residual blocks are used to extract image features in the encoder structure, which consists of 3 × 3 convolutional units and multiscale deep convolution (MSDC) with residual connectivity to the input feature mapping, and finally utilized after ReLu activation function. Each multiscale residual module is followed by a downsampling operation using the Haar wavelet transform, to reduce the size of the feature map, increase the number of channels, reduce computation, and extract more abstract feature information. After four downsampling operations, the size of the feature map is 16 × 16 × 256, which is relatively small in size and has a relatively large number of channels, contains global and local information of the input image, and provides a rich feature representation for the decoder. The feature map obtained by the encoder is further processed by the decoder for feature recovery. An upsampling operation is performed with transposed convolution to reduce the feature channels, The feature maps of the corresponding layers in the encoder are concatenated and fused using skip connections. The fused feature maps are subjected to triple attention computation and then further feature extraction is done using the multi-scale residual block. After four upsampling, the feature map with the same size as the input features is obtained, and finally the segmentation result map with the size of 256 × 256 × 1 is output after a 1 × 1 convolutional layer.

### 2.3. Multi-Scale Residual Block

**Figure 3:**
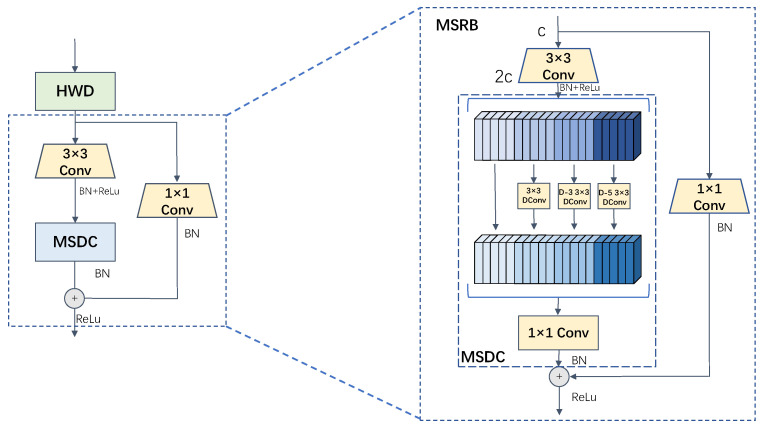
Structural diagram of the multiscale residual module, where DCConv denotes deep convolution, and D-3 and D-5 denote null rates of 3 and 5, respectively, Conv denotes convolution, + denotes an addition operation.

Various levels of contextual information are crucial for enhancing the accuracy of semantic segmentation [27]. This information aids in providing a more thorough understanding of the environment and semantic details surrounding the target object. Additionally, it enhances feature representation, allowing the model to grasp more intricate semantic information and detailed features, ultimately boosting the efficiency of feature extraction. Dilated convolution is a key method for acquiring multi-scale information, as it involves introducing a dilation rate to expand the receptive field of the convolution, distinguishing it from standard convolution techniques. In order to facilitate the extraction of multi-scale feature information, this research has restructured the network design of U-Net and introduced a more effective approach for acquiring such information. The improved network structure is illustrated in Figure 3. Each component of U-Net incorporates a residual design, allowing for quicker transfer of features from lower to higher layers, thereby enhancing the efficiency of feature transfer and the capacity to capture detailed information and edges of the target object in semantic segmentation tasks. Within the residual structure, initial feature extraction is carried out using standard convolution, followed by multiple deep convolutions with varying dilation rates to extract features from diverse receptive fields and merge them. This technique reduces parameter count, enhances computational efficiency, and enables the extraction of multi-scale feature information.

Specifically, the input feature map is subjected to a 3 × 3 convolutional layer, batch normalization, and ReLu activation function computation for initial feature extraction, the channel dimensions are raised to twice their original dimensions. The obtained feature maps are then equally divided into four parts, and the first part of the features are left unprocessed as a feature reuse layer. The second, third, and fourth parts are computed in parallel using deep convolution with a dilation rate of 1, 3, and 5, respectively, to obtain feature maps with different sensory field information, which are then spliced and fused and subjected to batch normalization operations. Finally, the spliced feature maps are fused using point-by-point convolution and residual concatenation with the input feature mapping to obtain a more comprehensive multi-scale feature representation [28].

### 2.4. Haar Wavelet Transform Downsampling

In convolutional neural networks, downsampling techniques are commonly utilized to decrease the resolution of feature maps. This helps to reduce the computational load of the network while also enhancing the model’s sensory field by aggregating local features. Downsampling is typically achieved through convolutional or pooling operations. While downsampling with convolution using a stride greater than 1 can improve feature representation, it is more computationally intensive compared to pooling methods. Pooling, such as maximum pooling and average pooling, is often used for semantic segmentation tasks due to its computational efficiency. However, pooling may discard important detailed information like texture and edge details. To address this issue and maintain efficiency, this study explores replacing maximum pooling with Haar Wavelet Transform-based downsampling [29] in the U-Net semantic segmentation architecture to retain more detailed information after downsampling.

**Figure 4:**
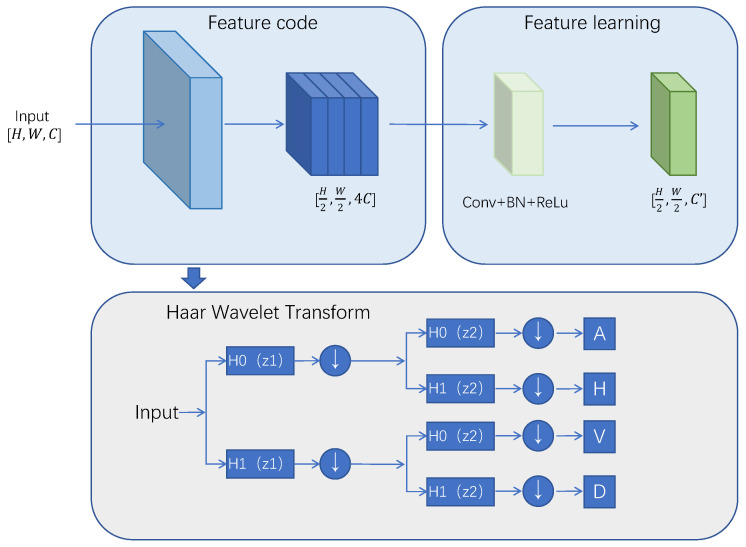
Haar Wavelet Downsampling structure, It mainly consists of a feature encoding module and a feature learning module. The feature encoding module is used to downsample the feature map, the feature learning module is used to adjust the number of channels of the feature map.

The downsampling process based on Haar Wavelet Transform (HWD) involves two main components: a feature encoding module and a feature learning module. The structure is shown in Figure 4. The feature encoding module utilizes Haar wavelet transform to decrease the resolution of the feature map while preserving detailed information. This transformation involves extracting low-frequency and high-frequency information from the image using low-pass (H0) and high-pass (H1) filters. The extracted information is then downsampled twice, resulting in the decomposition of the H × W image into four components: the low-frequency component (*A*), horizontal component (*H*), vertical component (*V*), and diagonal component (*D*), each of size H/2×H/2. These components are fused to create a final feature map without losing any information. The feature learning module includes a 1 × 1 convolutional layer, batch normalization, and ReLu activation function to adjust the channel numbers in the downsampled feature map, filter out unnecessary information, and enhance the learning of feature representations by the convolutional layer. The HWD-based downsampling method reduces the size of the H×W×C feature map to H/2, W/2 dimensions through feature encoding and learning modules. This method requires fewer parameters and computational resources compared to other downsampling techniques, effectively balancing the trade-off between computational efficiency and information loss.

### 2.5. Triple Attention Mechanism

**Figure 5:**
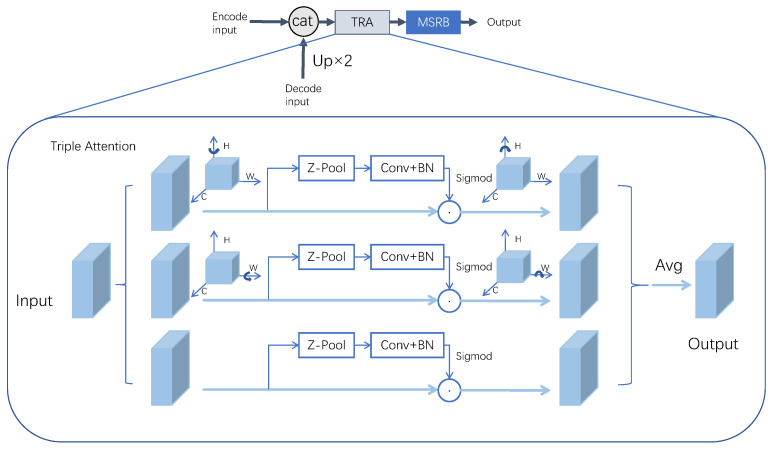
Structure of the triple attention mechanism with three branching structures, the first branching structure is used to compute the attention weights of the channel dimension C and the spatial dimension H, the second branching is used to compute the attention weights of the channel dimension C and the spatial dimension W, and the third branching is used to compute the attention weights of the spatial dimensions H and W. The details are shown below.

The attention mechanism has been widely used in the field of computer vision, which can adaptively adjust the weights according to the importance of different positions in the feature map, to pay more attention to the important features and suppress irrelevant information, improve the representation ability of the convolutional neural network without adding too many parameters. In this paper, we study the incorporation of a triple attention module [30] into the decoder structure in U-Net networks, which is less computationally intensive compared to attention such as CBAM [31], and achieves cross-dimensional information interaction without dimensionality reduction. Its network structure is shown in Figure 5. The input feature map needs to go through three branching structures to output the results, The first and second branches are responsible for establishing attention between the channel dimension C and the spatial dimensions W and H, respectively, the third branch is used to establish attention between the spatial dimensions H and W. Finally, the outputs of the three branches are fused using the mean value.

In the Figure 5, the Z-pool calculation is to compute the maximum pooling and average pooling of the 0th dimension of the tensor, The computed results are spliced to reduce their dimensions to two dimensions. This operation enables the tensor to retain rich feature information and reduces the computational effort, which can be expressed by the following equation:

(1) $$Z-pool\left(x\right)=[Maxpool_{0d}\left(x\right),Avgpool_{0d}\left(x\right)]$$

where 0*d* denotes the 0th dimension of the tensor.

For the input tensor of size C×H×W, the interaction between the channel dimension *C* and the height dimension *H* is established in the first branch, first, the input tensor *X* is rotated 90° counterclockwise by the H-axis to obtain the tensor ${\hat{X}}_{1}$ of shape size W×H×C, ${\hat{X}}_{1}$ undergoes a Z-pool calculation to obtain the 2×H×C tensor ${\hat{X}}_{1}^{*}$. Then convolution, batch normalization, and Sigmoid activation function computation are performed to generate the attention weights and multiply it by the input ${\hat{X}}_{1}$. The final tensor is rotated 90° clockwise along the H-axis and returns to the initial shape.

The second branch establishes the interaction between the channel dimension *C* and the width dimension *W*. The input tensor *X* is rotated 90° counterclockwise by the W-axis to obtain the tensor ${\hat{X}}_{2}$ with the shape size H×C×W. ${\hat{X}}_{2}$ undergoes the Z-pool computation to obtain the tensor ${\hat{X}}_{2}^{*}$ with 2×C×W. Then convolution, batch normalization, and Sigmoid activation function computation are performed to generate the attention weights, which are multiplied with the input ${\hat{X}}_{2}$, The tensor is eventually rotated 90° clockwise along the W axis to return to its initial shape.

The third branch is used to establish the spatial attention, the input tensor *X* is computed by Z-pool to get the tensor $\hat{X_{3}}$, whose shape size becomes 2×H×W, then convolution, batch normalization, Sigmoid activation function computation is carried out to generate the attention weights and then multiply them with the inputs to get the output results, finally the outputs of the three branches are summed up and taken as the mean value to get the output features. The calculation process can be represented by the following equation. The calculation process can be represented by the following equation.

(2) $$y=\frac{1}{3}\left({\hat{X}}_{1}\sigma \left(C_{1}\left({\hat{X}}_{1}^{*}\right)\right)+{\hat{X}}_{2}\sigma \left(C_{2}\left({\hat{X}}_{2}^{*}\right)\right)+X\sigma \left(C_{3}\left(\hat{X_{3}}\right)\right)\right)$$

where σ denotes the Sigmoid activation function, C denotes the convolutional computation.

### 2.6. Loss Function

In the infrared small target segmentation task belongs to the binary classification task, in this paper, we study the use of the Binary Cross Entropy loss function to train and optimize the model. Each pixel in the infrared image is labeled as a target or background, calculate the difference between the predicted and true value of each pixel using the binary cross-entropy loss function, and a weighted average of these errors, get the loss value for the whole image, which is used to measure the accuracy of the model’s prediction and as an optimization objective for the model. Adjustment of model parameters by gradient descent algorithm, Minimizing the loss value makes the predicted and true values as close as possible, thus improving the model performance. The formula is shown below.

(3) $$BCELoss=-\frac{1}{n}\sum_{i=1}^{n}\left[y_{i}log(p\left(y_{i}\right))+\left(1-y_{i}\right)log(1-p\left(y_{i}\right)\right)]$$

where $y_{i}$ denotes the label value of the *i*th sample, usually 0 or 1, and $p\left(y_{i}\right)$ denotes the predicted value of the model for the *i*th sample, i.e., the probability that the model predicts the label value of the *i*th sample to be 1. *n* is the total number of samples. When the label $y_{i}$ is 1, $BCELoss=-log\left(p\left(y_{i}\right)\right)$, if the predicted value $p\left(y_{i}\right)$ is close to 1, then the overall loss value is close to 0. Conversely, if the predicted value $p\left(y_{i}\right)$ is close to 0, then the overall loss value tends to positive infinity. When the label $y_{i}$ is 0, $BCELoss=-log\left(1-p\left(y_{i}\right)\right)$, if the predicted value $p\left(y_{i}\right)$ is close to 0, then the overall loss value is also close to 0. Predicted value $p\left(y_{i}\right)$ is close to 1, the loss value tends to positive infinity.

## 3. Experimental Analysis

### 3.1. Experimental Environment

During the experiment, the same parameters are set for experimental comparison, Adagrad is used as the optimizer, the initial learning rate is set to 0.05, and cosine annealing is used to adjust the learning rate during the training process. A total of 300 epoch is trained and Batch size is set to 8. The equipment used in this experiment is shown in Table 1.

### 3.2. Evaluation Metrics

In this paper, IoU, nIoU, Pd, Fa, ROC curve, and P-R curve metrics are used to evaluate the performance of the infrared small target detection algorithm. Where TP, FP, TN, and FN are true examples, false positive examples, true negative examples, and false negative examples, respectively, and T and P denote true and positive examples, respectively. Intersection-over-union (IoU) pixel-level evaluation metrics are commonly used in semantic segmentation tasks, It is used to evaluate the contour description ability of the algorithm, which is calculated as the ratio of the intersection region and the concatenation region of the predicted and real values, the formula is shown below:

(4) $$IoU=\frac{TP}{T+P-TP}$$

Normalized Intersection over Union (nIoU) is an evaluation metric specifically designed to measure IR small target detection. It is the arithmetic mean of each sample IoU, its calculation formula is shown below:

(5) $$nIoU=\frac{1}{n}\sum_{i=1}^{n}\frac{TP_{i}}{T_{i}+P_{i}-TP_{i}}$$

Precision indicates the ratio of true targets to all samples detected as targets, Probability of Detection (Pd), also known as Recall, is used to indicate the ratio of detected true targets to all true targets. The P-R curve with Precision as the vertical axis and Recall as the horizontal axis reflects the relationship between Precision and Recall, and usually the closer the curve is to the upper right the better the detection performance of the algorithm. The calculation formula is:

(6) $$Pd=\frac{TP}{TP+FN}$$

False-alarm rate (Fa) is used to indicate the ratio of wrongly predicted pixels to all pixels, the ROC curve with detection rate as vertical coordinate and false-alarm rate as horizontal coordinate, i.e., the subject’s work characteristics, is used to describe the relationship between the change of the detection rate and the false-alarm rate at different thresholds, it can effectively respond to the total effect of the algorithm under the sliding threshold. The calculation formula is:

(7) $$Fa=\frac{FP}{FP+TN}$$

### 3.3. Analysis of Ablation Experiments

To evaluate the performance of the network structure proposed in this paper, we use U-Net as the baseline network, Ablation experiments were performed on the test set in the NUDT-SIRST data. The experimental results are shown in Table 2.

In the table, the experiments test the effect of residual connectivity, Haar wavelet transform downsampling (HWD), Multiscale residual block (MSRB), and triple attention mechanism(TRA) on the network performance improvement, respectively. In particular, residual connectivity improved IoU and nIoU by 3.87% and 2.18%, respectively, Precision (−0.16%) showed a slight decrease, Recall (+1.08%) and F1-Score (+0.46%) were improved, and Fa (−0.7069 × $10^{-6}$) was decreased. This suggests that residual linking can improve the overall performance, but because residual linking adds shallow features directly to deep features may introduce some noise or interference, which has a slight effect on the accuracy of the network model. Replacing maximum pooling downsampling with HWD downsampling in U-Net’s encoder improves IoU and nIoU by 2.04% and 0.71%, respectively, Precision (+0.57%), Recall (+0.81%), and F1-Score (+0.69%), and decreases Fa (−0.0775 × $10^{-6}$). In addition, combining the residual connection and HWD downsampling can make the network performance better. IoU (+5.74%), nIoU (+3.43%), Precision (+0.83%), Recall (+1.48%), and F1-Score (+1.15%) are all greatly improved compared with U-Net, and Fa (−1.3247 × $10^{-6}$) decreases significantly, thus verifying that HWD downsampling is effective in reducing the information loss, combining with residual connectivity improves the information transfer efficiency, enhances the network’s ability to characterize the contours of small targets as well as its detection performance. Adopting the MSRB module for each structural unit of U-Net separately on this basis, the effect on IoU and nIoU was significantly improved, rising by 1.18% and 2.29%, respectively, the Precision increased by only 0.02%, Recall improved by 0.69%, Fa decreased by 0.7873 × $10^{-6}$, and F1-Score improved by 0.36%. Adding the triple attention mechanism(TRA) after decoder feature fusion increased IoU by 0.86%, nIoU by 0.55%, Precision by 0.23%, Recall by 0.14%, Fa by 0.115 × $10^{-6}$, and F1-Score by 0.18%. It is proved that MSRB with triple attention mechanism (TRA) plays an important role in the improvement of segmentation performance, which can effectively reduce the number of misdetected samples and make the target segmentation better.

### 3.4. Comparative Analysis of Multiple Methods

To evaluate the performance of the MST-UNet proposed in this paper in infrared small target segmentation, experimental comparisons with U-Net [32], ResUnet [33], ResUnet++ [34], UCTransNet [35], U-Net3+ [36], and DeepLab3+ [37] are analyzed in this section. The results are shown in Table 3. The IoU of MST-UNet proposed in this paper is 80.09%, nIoU is 80.19%, Precision is 98.75%, Recall is 98.51%, Fa is 1.2011 × $10^{-6}$, and F1-Score is 98.62%, which are better than other algorithms. ResUnet and ResUnet++, although based on an improved version of U-Net, are far inferior to U-Net in terms of various performance metrics, and thus the structural design of these two algorithms is not entirely appropriate for small target segmentation tasks. UCTransNet modifies the jump connections of U-Net into self-attention-based jump connections, and compared to U-Net, the false alarm rate (−1.6954 × $10^{-6}$) has a significant reduction, but performs poorly in terms of IoU (−0.67%), nIoU (−0.94%), Recall (−1.09%), and is slightly lower than that of U-Net, which suggests that the jump-connection direct connection approach is more suitable for small target segmentation than the self-attention approach. U-Net3+ utilizes multiscale jump connections to fuse multiscale information and outperforms U-Net in small target segmentation as a whole, with higher IoU (+2.29%), nIoU (+1.38%), and Recall (+0.13%), but not as good as U-Net in terms of Fa (+0.5288 × $10^{-6}$). This indicates that multiscale information fusion can significantly improve the effect of small target segmentation. DeepLab3+ is not effective for small target segmentation, with IoU and nIoU of 50.15% and 47.68%, respectively, and the other metrics of Recall (−9.34) and Fa (+1.7788 × $10^{-6}$) are all far from U-Net, so the algorithm’s suitability for small targets is not as good as the U-Net family of algorithms. Taken together, the MST-UNet proposed in this paper achieves the best results in all the metrics in the test set.

**Figure 6:**
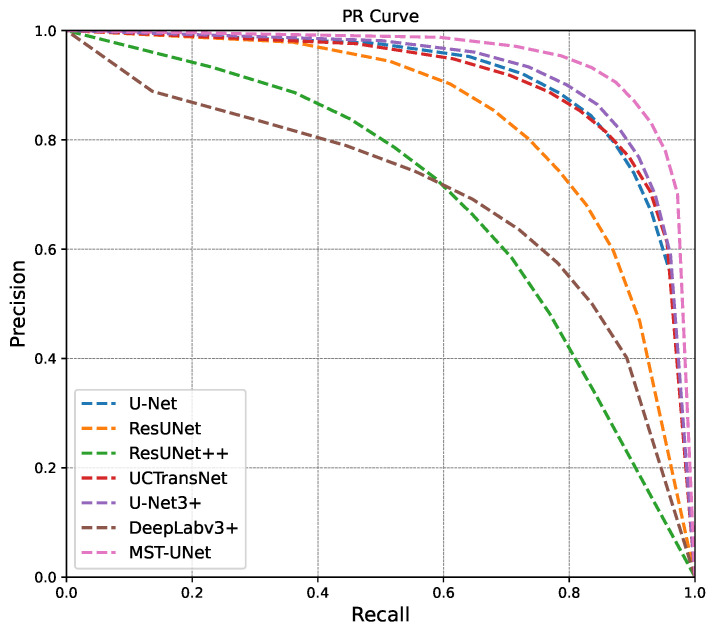
P-R Curve Comparison Chart.

**Figure 7:**
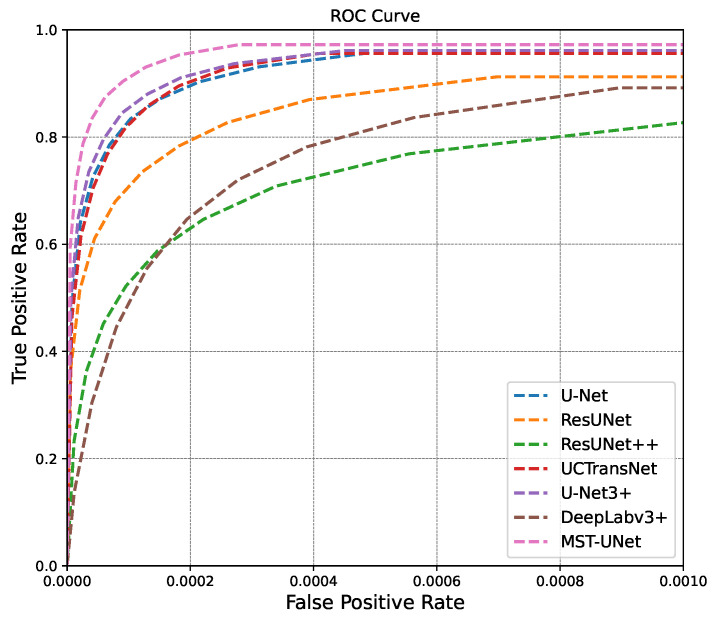
ROC Curve Comparison Chart.

Figure 6 and Figure 7 display the results of the performance evaluation for the P-R curve and ROC curve. The MST-UNet model introduced in this study outperforms other segmentation network models. The P-R curve, depicted in Figure 6, illustrates that the proposed method achieves superior precision and recall compared to other methods across various thresholds. The area under the curve for the algorithm in this paper is higher than that of other methods. The ROC curve, shown in Figure 7, demonstrates that the method in this paper consistently maintains optimal performance as the false alarm rate and Probability of Detection vary with different thresholds.

**Figure 8:**
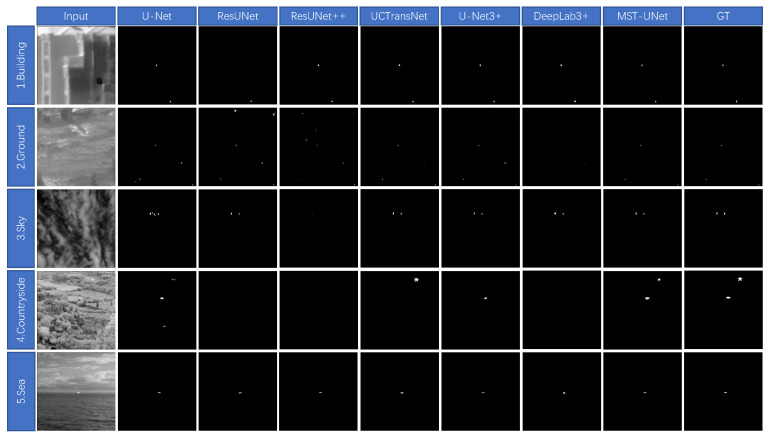
Segmentation result map from background 1 to background 5.

**Figure 9:**
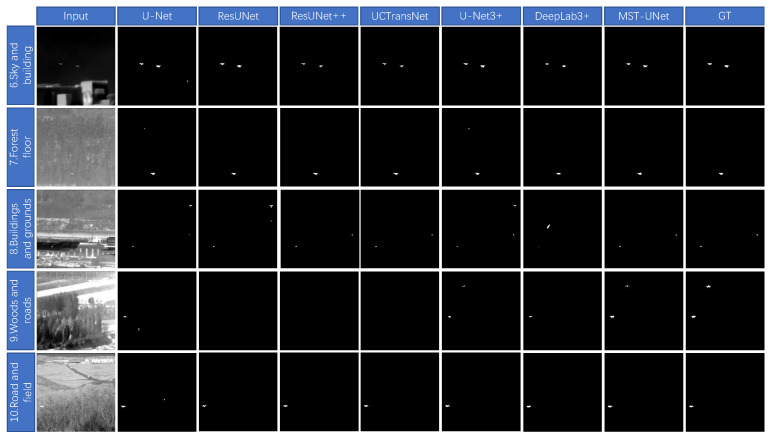
Segmentation result map from background 6 to background 10.

In this paper, the segmentation result graphs of each algorithm are compared, as shown in Figure 8 and Figure 9. Ten representative different backgrounds in the NUST-SIRST dataset are selected for testing respectively. As can be seen in Figure 9, background 1 is the building, ResUnet has a missed detection, and all other algorithms segment the target points correctly. Background 2 is the ground, U-Net has a small number of misdetected targets, ResUnet and ResUnet++ have a high number of misdetections, UCTransNet and U-Net3+ both have one misdetected target, DeepLab3+ does not detect the target, the algorithm proposed in this paper correctly segments the target. Background 3 is the sky and there are light clouds, U-Net has misdetections, ResUnet++ detects only one target point and the pixel description is too different from the real target.ResUnet, UCTransNet, U-Net3+, DeepLab3+, and the algorithms in this paper all correctly detect the target. Background 4 is a countryside, all algorithms perform poorly due to the low contrast between the target and the background, U-Net only detects one target and has two false detections, ResUnet, ResUnet++, and DeepLab3+ do not detect the target, UCTransNet and U-Net3+ only detect one target, this paper’s proposed method segmentation The effect is optimal and both targets are accurately segmented. Background 5 is the sea, There are no excessive clutter signals, so all algorithms segment the target correctly, the method in this paper achieves the best results in terms of contour description of the target.

In Figure 9, background 6 is the night sky and building background, U-Net has misdetection in the segmentation result, all other algorithms segment the target accurately, the proposed algorithms and U-Net3+ have the best results in the target contour description. Background 7 is the forest floor, U-Net, ResUnet++, and U-Net3+ have misdetection in different places, other algorithms and the proposed algorithm in this paper accurately segmented the target. There are scenes such as buildings and grounds in background 8, there are two targets in the figure, accompanied by more high-frequency noise, U-Net, ResUnet, U-Net3+ have been misdetected, ResUnet++ and DeepLab3 + only segmented to a target point, with a large difference in the pixel scale of the real target, DeepLab3 + also appeared to be a large chunk of misdetected phenomenon, only the UCTransNet and the proposed method in this paper correctly segmented in addition to the target. Background 9 In addition to some woods, there exists a large area of high-frequency road background, the target signal is not strong, which has a large impact on the segmentation results, so the three methods of ResUnet, ResUnet++, and UCTransNet do not segment the target, U-Net only correctly segments a target and there is a misdetected target, DeepLab3+ also has only one target, U-Net3 + and the methods in this paper segmented the target accurately, but the target contour is not continuous enough and more details are lost. Background 10 is the road field background, the target is more obvious, U-Net has one misdetection, the rest of the methods accurately locate the target, for the target contour description, ResUnet and DeepLab3+ have a large gap, and this paper’s method is the most effective. Taken together, this paper’s method performs superiorly in the small target segmentation task, proving the effectiveness of the method.

## 4. Conclusions

This paper focuses on enhancing the U-Net small target segmentation method by incorporating multi-scale feature fusion, decomposition, and an attention mechanism. The effectiveness of these enhancements is tested on the NUDT-SIRST dataset through various comparison experiments. The study introduces a novel approach to downsampling using the Haar wavelet transform instead of pooling to minimize information loss and improve feature map expression. Additionally, a multi-scale residual module is proposed to enhance the sensory field and gather multi-scale contextual information through dilated convolution. The feature representation is further refined by integrating a triple attention mechanism in the decoder to improve infrared small target segmentation. The proposed method is validated on the NUDT-SIRST dataset, showcasing the impact of Haar wavelet transform downsampling, multi-scale residual blocks, and the attention mechanism on small target segmentation through ablation experiments. Comparing and analyzing with algorithms such as U-Net, ResUnet, ResUnet++, UCTransNet, U-Net3+, DeepLab3+, the proposed method in this paper achieves the best results in several indexes, with IoU of 80.09%, nIoU of 80.19%, Pd of 98.51%, Fa of 1.2011 × $10^{-6}$, with good segmentation results for infrared small targets in different complex backgrounds.

## Figures and Tables

**Table 1:** Development environment.

Platform	Configuration
Integrated development environment	PyCharm
Deep Learning Framework	Pytorch
Scripting language	Python3.9
Operating system	Windows11
CPU	I5-12400F
GPU	NVIDIA GeForce RTX3060
Memory	16G
CUDA	11.7

**Table 2:** The effectiveness of the HWD, Res, MSRB, and TRA modules were analyzed in ablation experiments on the NUDT-SIRST dataset, respectively, the results are validated using four metrics: IoU, nIoU, Pd, and Fa.

NUDT-SIRST							
**Method**				**Metrics**			
**HWD**	**Res**	**MSRB**	**TA**	**IoU**	**nIoU**	**Pd**	**Fa(** $\times 10^{-6}$ **)**
				72.31	73.92	96.21	3.4281
✓				74.35	74.63	97.02	3.3506
	✓			76.18	76.1	97.29	2.7212
✓	✓			78.05	77.35	97.69	2.1034
✓	✓	✓		79.23	79.64	98.37	1.3161
✓	✓	✓	✓	80.09	80.19	98.51	1.2011

**Table 3:** Comparative tests of different segmentation models on the NUDT-SIRST dataset, the results are validated using four metrics: IoU, nIoU, Pd, and Fa.

NUDT-SIRST				
**Method**	**Metrics**			
	**IoU**	**nIoU**	**Recall (Pd)**	**Fa (** $\times 10^{-6}$ **)**
U-Net	72.31	73.92	96.21	3.4281
ResUnet	62.21	63.92	90.12	6.2127
ResUnet++	48.24	49.12	83.76	6.6609
UCTransNet	71.64	72.98	95.12	1.7327
U-Net3+	74.60	75.3	96.34	3.9569
DeepLab3+	50.15	47.68	86.87	5.2069
Ours	80.09	80.19	98.51	1.2011

## Data Availability

The data used to support the results of this study are included in the article.

## Abbreviations

The following abbreviations are used in this manuscript:

ACM	Asymmetric Context Modulation
DBT	Detect Before Track
DNA-Net	Dense Nested Attention Network
HWD	Haar wavelet transform downsampling
ILNet	low-level network
MDvsFA-cGAN	conditional generative adversarial network
MSDC	multiscale deep convolution
MSRB	Multiscale residual block
TBD	Track Before Detect
